# Fermented and Germinated Processing Improved the Protective Effects of Foxtail Millet Whole Grain Against Dextran Sulfate Sodium-Induced Acute Ulcerative Colitis and Gut Microbiota Dysbiosis in C57BL/6 Mice

**DOI:** 10.3389/fnut.2021.694936

**Published:** 2021-07-29

**Authors:** Yuhan Zhang, Wei Liu, Di Zhang, Yanbing Yang, Xianshu Wang, Lingfei Li

**Affiliations:** ^1^College of Food Science and Technology, Yunnan Agricultural University, Kunming, China; ^2^Institute of Agro-Food Science and Technology, Shandong Academy of Agricultural Sciences, Jinan, China; ^3^Qilu Hospital, Shandong University, Jinan, China

**Keywords:** foxtail millet, fermentation, germination, gut microbiota, inflammatory bowel disease, mouse colitis model

## Abstract

This study investigated the effects of foxtail millet whole grain flours obtained through different processing methods on alleviating symptoms and gut microbiota dysbiosis in a dextran sulfate sodium (DSS)-induced murine colitis model. Sixty C57BL/6 mice were divided into six groups (*n* = 10 in each group), including one control group (CTRL) without DSS treatment and five DSS-treated groups receiving one of the following diets: AIN-93M standard diet (93MD), whole grain foxtail millet flour (FM), fermented (F-FM), germinated (G-FM), and fermented-germinated foxtail millet flour (FG-FM). A comparison of the disease activity index (DAI) demonstrated that foxtail millet whole grain-based diets could alleviate the symptoms of enteritis to varying degrees. In addition, 16S rRNA gene sequencing revealed that FG-FM almost completely alleviated DSS-induced dysbiosis. Mice on the FG-FM diet also had the lowest plasma IL-6 levels and *claudin2* expression levels in the colon, indicating reduced systemic inflammation and improved gut barrier function. This study suggested that foxtail millet whole grain is an attractive choice for the intervention of IBD and gut microbiota dysbiosis, and its prebiotic properties are highly affected by the processing methods.

## Introduction

Inflammatory bowel disease (IBD) is a chronic and incurable inflammatory disease that has two major entities: Crohn's disease (CD) and ulcerative colitis (UC) (1). Patients with IBD suffer from multiple clinical symptoms, including abdominal pain, diarrhea, blood in stools, and weight loss, as well as having a higher risk of developing colorectal carcinoma (CRC) (2). In this century, IBD has become a global health concern because of its high disease burden in Western countries and increasing prevalence in newly industrialized countries in Asia, Africa, and South America (3). At present, IBD is widely accepted to be a multifactor disease driven by complex interactions among genetics, environmental factors, and the host immune system (4). The precise etiology of IBD is still unclear, which is a major problem for the prevention and medical therapy of IBD (5).

The gut microbiota is a collective term for microorganisms that live in the intestine of humans and other animals. The gut microbiota is primarily made up of bacteria, but it also includes viruses, archaea, and fungi inhabiting the gastrointestinal tract (6). It is the largest independent organ of the human body, performing indispensable functions such as digestion, absorption, metabolism, and immunity (7). There has been increasing evidence indicating that dysbiosis of the gut microbiota, which is usually manifested as a decrease in gut microbial diversity or a shift in the balance between commensal and potentially pathogenic bacteria (1), plays an important role in IBD and IBD-associated CRC (6). The reduction in gut microbial diversity, as well as differences in the composition of gut microbiota between IBD patients and healthy controls, have previously been demonstrated (8). For instance, several species of the phylum *Proteobacteria* have been reported as microbial signatures for gut microbiota in IBD patients (9). The most studied member of the *Proteobacteria, Escherichia coli*, was found to be excessively proliferated in IBD patients, playing a non-eligible role in the development of IBD (10). Along with an increase in the relative abundance of species from the phylum *Proteobacteria*, the abundance of the phyla *Firmicutes* and *Bacteroidetes*, which are the dominant phyla in the gut microbiota of healthy individuals, was significantly decreased in IBD patients (11).

The key factors for the pathogenesis of IBD have been highlighted. Therefore, the gut microbiota has emerged as a promising new target for the prevention and clinical treatment of IBD. In clinical practice, the application of fecal microbiota transplantation (FMT) has been proven to be effective in alleviating clinical symptoms in IBD patients (12). However, the potential hazards of FMT remain controversial, making the possibility of its widespread application unclear (13). Probiotic therapy has also been shown to be effective in both murine colitis models and IBD patients (14). In addition, due to the direct impact on the gut microbiota (15), dietary ingredients can also be used as promising strategies for the prevention and therapy of IBD due to their direct impact on the gut microbiota (15). Several food polyphenols and specific carbohydrates, for example, are effective in relieving the clinical symptoms of IBD (16, 17).

Cereals are at the bottom of the nutrition pyramid and make up a large percentage of the daily diet. Foxtail millet (S*etaria italica Beauv*.) is an old staple crop in Europe and Asia and is still one of the main food crops in northern China (18). Foxtail millet has superior nutritional properties among cereals, containing high amounts of proteins, minerals, and vitamins (19). In addition to being a daily nutritional source, small millets, including foxtail millet, have anti-inflammatory properties and can therefore be used for the prevention of related chronic diseases, such as atherosclerosis and diabetes (20, 21). Along with its superior nutritional properties and documented anti-inflammatory function, foxtail millet is also rich in non-nutrient prebiotics, such as polyphenols and dietary fibers (22), suggesting its possible positive effects on the gut microbiota and IBD. Bond polyphenols and peroxidase of foxtail millet bran have also been reported to inhibit colitis-induced carcinogenesis in a mouse model (23, 24).

In daily life, the foxtail millet is usually peeled and refined rather than the whole grain. The dehulling process results in a significant decrease in antinutrients and an increase in the bioavailability of foxtail millet, reducing the burden on the digestive system (19). However, the cereal bran, which is lost during the peeling process, contains abundant prebiotics that can effectively promote the growth of probiotics, modulate the bacterial composition of gut microbiota, and increase the production of short-chain fatty acids (SCFAs) (25). In addition to dehulling, germination and fermentation have also been reported to decrease the contents of anti-nutrients and improve the digestibility of whole grains, such as millets (26). Germination induces a significant increase in free amino acids, as well as significant beneficial effects on the availability of polyphenolic components, minerals, and γ-aminobutyrate (GABA) in foxtail millet (27, 28). On the other hand, the fermentation process causes the degradation of cellulose and hemicellulose in the cereal bran, resulting in the formation of more porous and loose structures and polysaccharides, which significantly improves the digestibility and prebiotic properties of foxtail millet (26).

In order to develop cost-effective dietary strategies for the prevention of IBD, this study investigated the alleviation of gut microbiota dysbiosis and the reduction in the symptoms of DSS-induced murine colitis models using foxtail millet flours obtained through different processing methods. Based on the hypothesis that different pretreatment methods will affect the function of millet in the prevention of colon colitis, four foxtail millet whole-grain cereal flours were studied: foxtail millet whole grain flour (FM), fermented foxtail millet whole grain flour (F-FM), germinated foxtail millet whole grain flour (G-FM), and fermented-germinated foxtail millet flour (FG-FM). This study used a traditional staple food in daily life as a dietary intervention for IBD to provide a new perspective for the prevention and treatment of IBD.

## Materials and Methods

### Preparation of Foxtail Millet Cereal Flours and Animal Diets

The foxtail millet used in this study was provided by the Crop Institute, Shandong Academy of Agricultural Science (Jinan, China). Both FM and G-FM were prepared using foxtail millet whole grains and germinated foxtail millet whole grains, respectively. F-FM and FG-FM were prepared by fermenting foxtail millet whole grains or germinated foxtail millet whole grains by fermentation using *Lactobacillus plantarum* NBRC 15,891 (29), respectively. The following are the preparation details:

**FM:** The foxtail millet whole grain seeds were ground and screened using an 80-mesh sieve to prepare the foxtail millet whole grain flour (FM).

**F-FM:** The FM flour was mixed with tap water (1:3) and heated for 10 minutes in a 75±5°C water bath to gelatinize the starch. After that, it was inoculated with *L. plantarum* NBRC 15,891 at a concentration of 10^6^ CFU per 100 g FM flour and incubated at 37°C until the pH dropped to 4.0 ± 0.2. The resulting slurry was dried at 55°C, ground and screened using an 80-mesh sieve to produce fermented polished foxtail millet flour (F-PFM).

**G-FM:** The germinated foxtail millet was prepared as described previously (30). The foxtail millet seeds were soaked in tap water overnight at room temperature. After draining the water, the seeds were spread out on a moist muslin cloth and covered with moist absorbent gauze. The seeds were left to sprout at room temperature for 48 h. After sprouting, the germinated seeds were dried at 55°C before being ground and screened using an 80-mesh sieve to prepare the germinated whole-grain foxtail millet flour (G-FM).

**FG-FM:** FG-FM flour was prepared using G-FM flour following the same preparation procedure that was used for F-FM.

The contents of starch, total proteins, crude fats, ashes, dietary fibers, and the moisture of cereal flours are provided in (Supplementary Table 1). Cereal flour-based animal diets were designed based on the AIN-93M standard rodent formula. The foxtail millet test diets contained 50% cereal flours, with the remaining 50% supplemented with standard nutrients according to the AIN-93M formula. The animal diets were prepared by Nantong Troffe Technology Co., Ltd. (Jiangsu, China). The detailed compositions of the experimental diets are provided in (Supplementary Table 2).

### Detection of the Main Compounds in Foxtail Millet Cereal Flours With LC/MS Analysis

The detailed method of sample preparation has been previously described (31). A total of 50 mg sample was weighted, and a 1,000 μL extract solution (methanol: water = 3:1 with isotopically labeled internal standard mixture) was added. The samples were then homogenized at 35 Hz for 4 min and sonicated for 5 min in an ice-water bath. Both the homogenization and sonication cycles were repeated three times. After that, the samples were incubated for 1 h at −40 °C and centrifuged at 12,000 rpm for 15 min at 4 °C. The resulting supernatant was transferred to a new glass vial for analysis. The quality control (QC) sample was prepared by mixing an equal aliquot of the supernatants from each sample.

The LC-MS/MS analyses were performed using a UHPLC system (Vanquish, Thermo Fisher Scientific) with a UPLC HSS T3 column (2.1 × 100 mm, 1.8 μm) coupled to a Q Exactive HFX mass spectrometer (Orbitrap MS, Thermo). The mobile phase consisted of 5 mmol/L ammonium acetate and 5 mmol/L acetic acid in water (A) and acetonitrile (B). The auto-sampler temperature was 4 °C, and the injection volume was 3 μL. The QE HFX mass spectrometer was used to acquire MS/MS spectra using the information-dependent acquisition (IDA) mode in the control of the acquisition software (Xcalibur, Thermo). In this mode, the acquisition software continuously evaluates the full scan MS spectrum. The following ESI source parameters were used: sheath gas flow rate at 30 Arb, Aux gas flow rate at 10 Arb, capillary temperature at 350 °C, full MS resolution at 60,000, MS/MS resolution at 7,500, collision energy at 10/30/60 in NCE mode, and spray Voltage at 4.0 kV (positive) or −3.8 kV (negative), respectively. The resulting raw data were converted to the mzXML format using ProteoWizard and processed with an in-house program, which was developed using R and based on XCMS, for peak detection, extraction, alignment, and integration. Then, metabolite annotation was performed using an in-house MS2 database (BiotreeDB). The cutoff for annotation was set at 0.3.

### Animals Experiments Design

**Figure 1:**
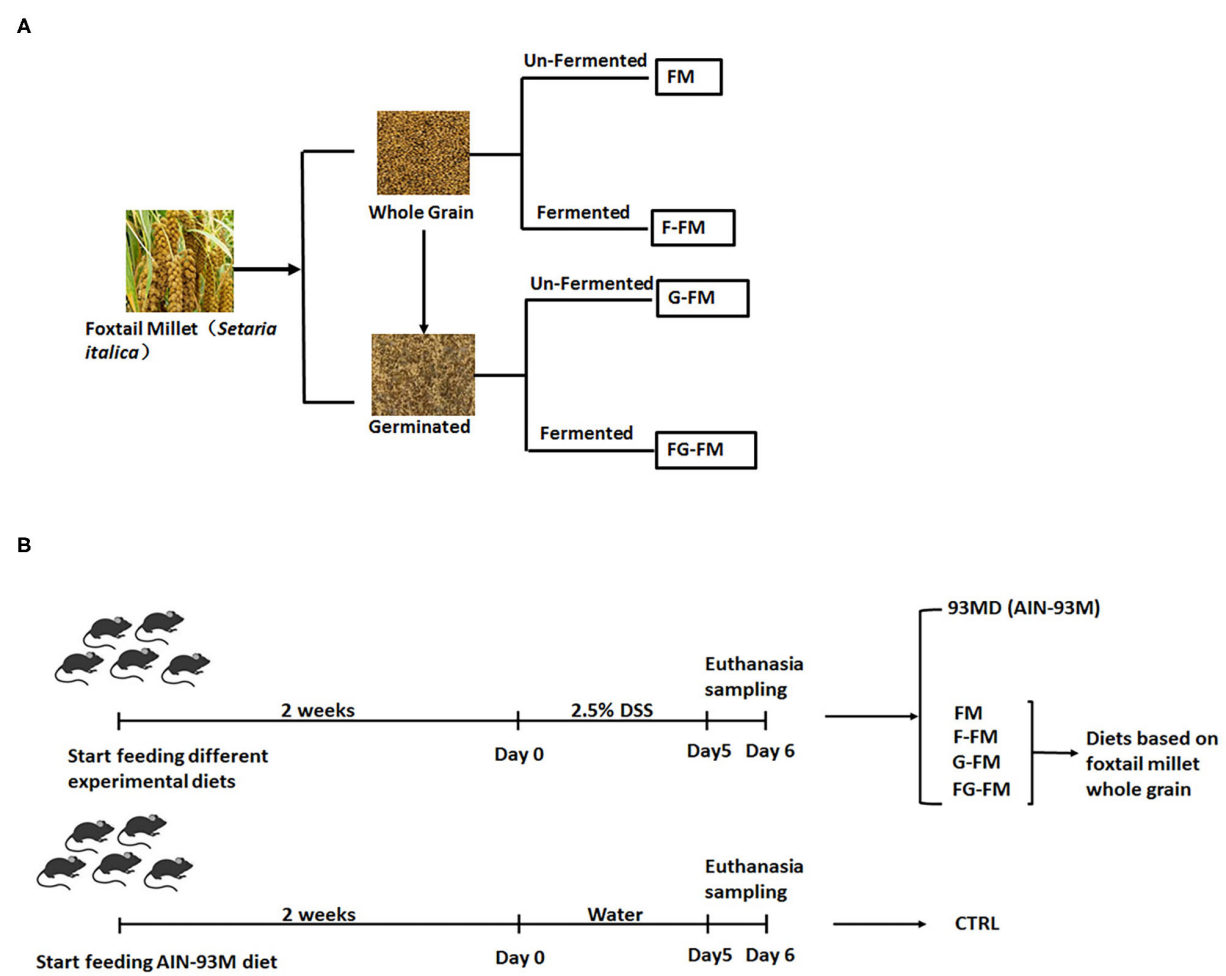
Experimental design illustration: **(A)** preparation of foxtail millet-based animal diets and **(B)** animal experimental protocol.

Preparation of foxtail millet-based animal diets and animal experimental protocols are shown in Figures 1A,B. Sixty male C57BL/6 mice (6 weeks old) were purchased from the Model Animal Research Center of Nanjing University (Nanjing, China) and housed in a specific pathogen-free facility with ad libitum access to food and water. After two weeks of acclimation, the mice were randomly assigned to one of the following six experimental diet groups (*n* = 10 per diet, 5 mice per cage): CTRL group (control group; mice fed an AIN-93M standard diet, without being exposed to DSS); 93MD group (mice fed an AIN-93M standard diet); FM group (mice fed an FM flour diet); F-FM group (mice fed an F-FM flour diet); G-FM group (mice fed a G-FM flour diet) and FG-FM group (mice fed an FG-FM flour diet).

After one week of experimental diet supplementation, all mice (except mice from the CTRL group) were exposed to DSS treatment (molecular weight 36–50 kDa; MP Biomedicals) in their drinking water (2.5%) for five consecutive days, with the first day of DSS treatment was designated as day 0. The DSS solution was freshly prepared and replaced every day. After five days of DSS treatments, the mice were anesthetized with isoflurane, and blood was collected with a capillary tube containing 1% heparin sodium solution by orbital puncture. Following that, the blood was centrifuged at 6,000 rpm for 10 minutes to obtain plasma. After the mice were sacrificed by cervical dislocation, their abdomens were opened and cecal contents were collected, snap-frozen in liquid nitrogen, then stored at −80°C for further analysis. The length of the mouse colon was measured from the colon-cecal junction to the anus. A 2-cm section of the mouse colon was fixed for histological analysis, and then the rest was collected and snap-frozen in liquid nitrogen for further analysis. The spleen and liver weights of the mice were recorded. The animals were cared for according to the Guide for P.R. China Legislation on the Use and Care of Laboratory Animals. All experimental procedures were approved by the Animal Care and Use Ethics Committee of the Institute of Agro-food Science and Technology at Shandong Academy of Agricultural Sciences (Jinan, China) (Approval ID: SDAA-IAFST-2018003).

### Evaluation of Disease Activity Index (DAI)

During induction, disease progression was evaluated by recording body weight, stool characteristics and rectal bleeding every day. The DAI was determined by combining the measured scores of body weight (0–4), stool consistency (0–3), and rectal bleeding (0–3) as previously described (32, 33). The measured score ranges were as follows: body weight loss (0 = ≤ 1%; 1 = 1–5%; 2 = 5–10%; 3 = 10–20%; and 4 = >20%), stool consistency (0 = normal; 1 = soft stools; 2 = loose stools; and 3 = diarrhea), and rectal bleeding (0 = negative hemoccult; 1 = hemoccult; 3 = traces of blood; and 4 = visible rectal bleeding).

### Histopathological Scores

For histopathological observations, the colon tissues were fixed in 10% formalin for more than 48 h, sectioned at 5 μm, and stained with hematoxylin-eosin. Eight fields were randomly selected in each section and were scored for colonic epithelial damage (0–6), inflammatory infiltrate in the mucosa (0–3), submucosa (0–2) and muscularis/serosa (0–1) as previously described (34). The given scores were described as follows: colonic epithelial damage (0 = normal; 1= hyperproliferation, irregular crypts, and goblet cell loss; 2 = 10–50% crypt loss; 3 = 50–90% crypt loss; 4 = complete crypt loss, surface epithelium intact; 5 = small- to medium-sized ulcer, <10 crypt width; 6 = large-scale ulcer, <10 crypt width), inflammatory infiltrate in the mucosa (0 = normal; 1 = mild; 2 = modest; 3 = severe), submucosa (0 = normal; 1 = mild to modest; 2 = severe), and muscularis/serosa (0 = normal; 1 = moderate to severe). The average scores of eight randomly selected fields for all four individual scores were added, which resulted in a total scoring range of 0–12 per colon.

### Analysis of Gut Microbiota Using 16S rRNA Gene Sequencing

The 16S rRNA gene sequencing method has been previously described (33). Total bacterial DNA was extracted from frozen cecal content, the V3-V4 region of the 16S rRNA gene was amplified, and a DNA library was constructed. Paired-end sequencing with a read length of 2 × 150 bp was performed using the Illumina HiSeq platform (Illumina, Inc., San Diego, California). The demultiplexed paired-end reads were joined using the FLASH v1.2.7 software (35), quality filtered (36), and the chimerism was removed (37). The resulting tags were then assigned to OTUs (38) with a 97% threshold of pairwise identity and aligned using the Silva reference database (Release 128, http://www.arb-silva.de) (39).

### Quantification of Inflammatory Cytokines in Mouse Plasma

Mouse plasma samples were centrifuged at 3,000 rpm for 10 minutes at 4°C, then the supernatant was collected and diluted 2-fold using the diluent provided in the Proinflammatory Panel 1 (mouse) V-PLEXTM Kit. The concentrations of IL-6 and IL-1β were measured using the V-PLEXTM mouse IL-6 Kit and V-PLEXTM mouse IL-1β Kit on a MESO QuickPlex SQ 120 (Mesoscale Discovery, Rockville, MD) according to the manufacturers' instructions.

### Gene Expression Analysis by qRT-PCR

The relative expression levels of genes related to intestinal barrier function (*Claudin1, Claudin2, ZO-1*, and *Occludin*) in the colon were determined using qRT-PCR. Total RNA was extracted from a 1-cm-long colon segment following the cecum using the TRIzol RNA extraction method (Invitrogen, Carlsbad, CA, USA) and quantified using a NanoDrop 2,000 spectrophotometer. The total quantified RNA was then reverse transcribed to cDNA using a QuantiTect Reverse Transcription Kit (Qiagen, USA). The relative expression levels of the selected genes were determined using the ViiA™ 7 Real-Time PCR System (Applied Biosystems, USA). The Ct values of each gene were first normalized to the housekeeping gene *GAPDH* (ΔCt value), and the fold-change in the same gene in the control sample (mice from the CTRL group) was calculated using the 2^−ΔΔCt^ method for mRNA quantification. The primer sequences for all the genes (*GAPDH, claudin 1, claudin 2, ZO-1*, and *occludin*) are provided in (Supplementary Table 3).

### Statistics

Data sets involving more than two groups were assessed using one-way ANOVA, followed by Fisher's LSD *post hoc* tests using GraphPad Prism version 6.00 for Windows (GraphPad Software Inc., USA). A value of *P* < 0.05 was considered to be statistically significant and presented as the mean ± SD. A heatmap was created based on transformed z-score values using the HemI software.

## Results

### Comparison of the Main Compounds of Differential Pre-treated Foxtail Millet Flours

The main compounds of Foxtail millet flours were identified using LC/MS. Comparisons with accurate mass and MS/MS data of natural products in MS2 databases revealed more than 1,000 main compounds in foxtail millet flours; In order to accurately identify more compounds influenced by different pre-treated methods, a strict filter condition was set (MS score > 0.99; VIP > 1). After filtering, the changes of the main compounds across the groups were compared, and compounds that significantly changed (*P* < 0.05) were listed in Tables 1–3.

**Table 1:** The significantly differential compounds in FM flour vs. F-FM flour.

**Name**	**FM (Mean ± SD)**	**F-FM (Mean ± SD)**	**Fold change**	***P*** **-Value**
Phosphoric acid	0.027 ± 0.0021	0.016 ± 0.0016	0.61	0.0022
Cytosine	0.0073 ± 0.00094	0.27 ± 0.021	37.40	0.0020
Hypoxanthine	0.19 ± 0.00099	0.34 ± 0.0072	1.82	<0.0001
Niacinamide	0.071 ± 0.0046	0.23 ± 0.0099	3.32	<0.0001
Adenosine	1.30 ± 0.029	0.21 ± 0.0043	0.16	0.00018
Choline	3.59 ± 0.59	8.29 ± 0.31	2.31	0.00025
D-Proline	0.71 ± 0.033	0.98 ± 0.013	1.37	0.00021
Pyrrolidine	0.0069 ± 0.0019	0.041 ± 0.00036	5.95	<0.0001
Yuccaol C	0.15 ± 0.013	0.28 ± 0.012	1.89	0.00022
2,5-Dihydro-2,4-dimethyloxazole	0.36± 0.016	0.52 ± 0.083	1.44	0.032
L-Serine	0.071 ± 0.0060	0.014 ± 0.0034	0.20	0.00014
Guanine	0.16 ± 0.012	2.55 ± 0.055	16.27	<0.0001
N2,N2-Dimethylguanosine	0.0063 ± 0.00036	0.018 ± 0.00066	2.83	<0.0001
Putrescine	0.016 ± 0.00037	0.019 ± 0.00052	1.19	0.0011
6-Angeloylfuranofukinol	0.0043 ± 0.00039	0.13 ± 0.0038	31.14	0.00025
N-Methyltyramine	2.09 ± 0.17	4.32 ± 0.093	2.06	<0.0001
(Â±)-erythro-Isoleucine	1.70 ± 0.051	0.089 ± 0.0078	0.052	0.00025
ent-Epiafzelechin(2a->7,4a->8)epiafzelechin 3-(4-hydroxybenzoic acid)	0.019 ± 0.00031	0.0090 ± 0.00053	0.48	<0.0001
11-Hydroxyeicosatetraenoate glyceryl ester	0.00090 ± 0.00014	0.00015 ± 0.000065	0.17	0.0012
L-Phenylalanine	1.11 ± 0.077	0.13 ± 0.011	0.12	0.0017
2-Furancarboxaldehyde	2.09 ± 0.072	0.15 ± 0.0054	0.072	0.00042
p-Aminobenzoic acid	0.40 ± 0.0085	0.35 ± 0.015	0.86	0.0052
Prostaglandin H2 2-glyceryl Ester	0.021 ± 0.0014	0.012 ± 0.00026	0.59	0.00049
Benzyl gentiobioside	0.0010 ± 0.00016	0.00054 ± 0.000040	0.51	0.0060
L-Threonine	0.034 ± 0.0034	0.026 ± 0.0027	0.75	0.027
Guanosine	0.087 ± 0.0019	0.007 8± 0.00050	0.090	<0.0001
13,14-Dihydro-15-keto PGF2a	0.0034 ± 0.00036	0.0051 ± 0.00029	1.50	0.0029

**Table 2:** The significantly differential compounds in FM flour vs. G-FM flour.

**Name**	**FM (Mean ± SD)**	**G-FM (Mean ± SD)**	**Fold change**	***P*** **-Value**
Phosphoric acid	0.027 ± 0.0021	0.054 ± 0.0029	2.03	0.00018
Niacinamide	0.071 ± 0.0046	0.23 ± 0.0076	3.29	<0.0001
Adenosine	1.30 ± 0.029	1.48 ± 0.058	1.14	0.0087
Choline	3.59 ± 0.59	8.90 ± 1.70	2.48	0.0070
D-Proline	0.71 ± 0.033	5.39 ± 0.54	7.56	0.0042
Pyrrolidine	0.0069 ± 0.0019	0.22 ± 0.083	32.51	0.045
Yuccaol C	0.15 ± 0.013	0.24 ± 0.024	1.67	0.0035
2,5-Dihydro-2,4-dimethyloxazole	0.36 ± 0.016	0.53 ± 0.10	1.47	0.044
L-Serine	0.071 ±± 0.0060	0.24 ± 0.046	3.47	0.021
N2,N2-Dimethylguanosine	0.0063 ± 0.00036	0.0019 ± 0.00064	0.30	0.00049
Avocadyne	0.022 ± 0.00029	0.013 ± 0.000081	5.67	<0.0001
2-Pyrrolidinone	0.051 ± 0.0021	0.060 ± 0.0052	1.18	0.044
Putrescine	0.016 ± 0.00037	0.10 ± 0.021	6.38	0.020
6-Angeloylfuranofukinol	0.0043 ± 0.00039	0.012 ± 0.00055	2.71	<0.0001
N-Methyltyramine	2.09 ± 0.17	4.09 ± 0.063	1.95	<0.0001
(Â±)-erythro-Isoleucine	1.70 ± 0.051	18.47 ± 1.69	10.85	0.0034
ent-Epiafzelechin(2a->7,4a->8)epiafzelechin 3-(4-hydroxybenzoic acid)	0.019 ± 0.00031	0.0092 ± 0.0025	0.49	0.021
2-[4-(3-Hydroxypropyl)-2-methoxyphenoxy]-1,3-propanediol 1-xyloside	0.0012 ± 0.00011	0.0016 ± 0.00013	1.41	0.0084
11-Hydroxyeicosatetraenoate glyceryl ester	0.00090 ± 0.00014	0.0063 ± 0.00060	7.06	0.00011
Quinoline	0.0079 ± 0.0011	0.033 ± 0.0020	4.21	<0.0001
Oxolan-3-one	0.043 ± 0.0023	0.050 ± 0.00082	1.16	0.0071
L-Phenylalanine	1.11 ± 0.077	14.44 ± 0.44	13.01	<0.0001
2-Furancarboxaldehyde	2.09 ± 0.072	3.59 ± 0.31	1.72	0.0012
Maltotetraose	0.00056 ± 0.00012	0.0025 ± 0.000031	4.53	0.00050
Piperidine	0.016 ± 0.0018	0.023 ± 0.0020	1.45	0.010
Benzyl gentiobioside	0.0010 ± 0.00016	0.046 ± 0.0048	43.46	0.0038
L-Threonine	0.034 ± 0.0034	0.46 ± 0.042	13.48	0.0031
Guanosine	0.087 ± 0.0019	0.17 ± 0.0038	1.91	<0.0001
13,14-Dihydro-15-keto PGF2a	0.0034 ± 0.00036	0.0016 ± 0.00011	0.48	0.0012

**Table 3:** The significantly differential compounds in FM flour vs. FG-FM flour.

**Name**	**FM (Mean ± SD)**	**FG-FM (Mean ± SD)**	**Fold change**	***P*** **-Value**
Phosphoric acid	0.027 ± 0.0021	0.12 ± 0.0058	4.41	<0.0001
Cytosine	0.0073 ± 0.00094	0.63 ± 0.030	87.18	0.00078
Hypoxanthine	0.19 ± 0.00099	1.12 ± 0.016	5.95	<0.0001
Niacinamide	0.071 ± 0.0046	0.55 ± 0.010	7.75	<0.0001
Adenosine	1.30 ± 0.029	0.50 ± 0.014	0.38	<0.0001
Thiamine	0.086 ± 0.027	0.036 ± 0.0025	0.42	0.088
Etonogestrel	0.0042 ± 0.00069	0.019 ± 0.0011	4.55	<0.0001
D-Proline	0.71 ± 0.033	6.46 ± 0.27	9.07	0.00064
Pyrrolidine	0.0069 ± 0.0019	0.22 ± 0.054	31.50	0.021
Yuccaol C	0.15 ± 0.013	0.35 ± 0.092	2.43	0.056
L-Serine	0.071 ± 0.0060	0.0012 ± 0.00031	0.017	0.0024
Guanine	0.16 ± 0.012	6.81 ± 0.18	43.46	0.00024
N2,N2-Dimethylguanosine	0.0063 ± 0.00036	0.020 ± 0.00074	3.13	<0.0001
Avocadyne	0.022 ± 0.00029	0.0093 ± 0.00078	4.12	0.00013
Putrescine	0.016 ± 0.00037	0.070 ± 0.014	4.35	0.021
6-Angeloylfuranofukinol	0.0043 ± 0.00039	0.093 ± 0.0024	21.54	0.00018
N-Methyltyramine	2.09 ± 0.17	5.17 ± 0.097	2.47	<0.0001
(Â±)-erythro-Isoleucine	1.70 ± 0.051	8.24 ± 0.66	4.84	0.0033
ent-Epiafzelechin(2a->7,4a->8)epiafzelechin 3-(4-hydroxybenzoic acid)	0.019 ± 0.00031	0.0014 ± 0.00036	0.074	<0.0001
2-[4-(3-Hydroxypropyl)-2-methoxyphenoxy]-1,3-propanediol 1-xyloside	0.0012 ± 0.00011	0.0015 ± 0.000083	1.30	0.012
Quinoline	0.0079 ± 0.0011	0.020 ± 0.0014	2.58	0.00027
L-Phenylalanine	1.11 ± 0.077	5.18 ± 0.12	4.67	<0.0001
2-Furancarboxaldehyde	2.09 ± 0.072	4.08 ± 0.20	1.96	<0.0001
Maltotetraose	0.00056 ± 0.00012	0.0085 ± 0.00035	15.10	<0.0001
Benzyl gentiobioside	0.0010 ± 0.00016	0.032 ± 0.00057	31.02	<0.0001
L-Threonine	0.034 ± 0.0034	0.083 ± 0.010	2.43	0.0015
Guanosine	0.087 ± 0.0019	0.032 ± 0.00086	0.37	<0.0001
13,14-Dihydro-15-keto PGF2a	0.0034 ± 0.00036	0.0014 ± 0.00017	0.40	0.00082
L-Valine	0.21 ± 0.0083	0.13 ± 0.0029	0.62	<0.0001

As shown in Tables 1–3, when comparing the effects of differential pre-treatment methods (fermentation, germination, or both) on the main compounds in foxtail millet whole grain (F-FM vs. FM; G-FM vs. FM; FG-FM vs. FM), 27 deferentially expressed compounds were identified in F-FM (compared to FM); 29 deferentially expressed compounds were identified in G-FM (compared to FM); 29 deferentially expressed compounds were identified in FG-FM (compared to FM). It was worth noting that 19 compounds had significant changes in all three comparisons (F-FM vs. FM; G-FM vs. FM; FG-FM vs. FM). Out of these 19 compounds, 8 of them, including Niacinamide, D-Proline, Pyrrolidine, Yuccaol C, Putrescine, N-Methyltyramine, 2-[4-(3-Hydroxypropyl)-2-methoxyphenoxy]-1,3-propanediol 1-xyloside, and Benzyl gentiobioside were up-regulated in all three pre-treated foxtail millets (F-FM, G-FM, FG-FM) when compared to un-treated foxtail millet whole grain (FM).

### Body Weight Loss and DAI

**Figure 2:**
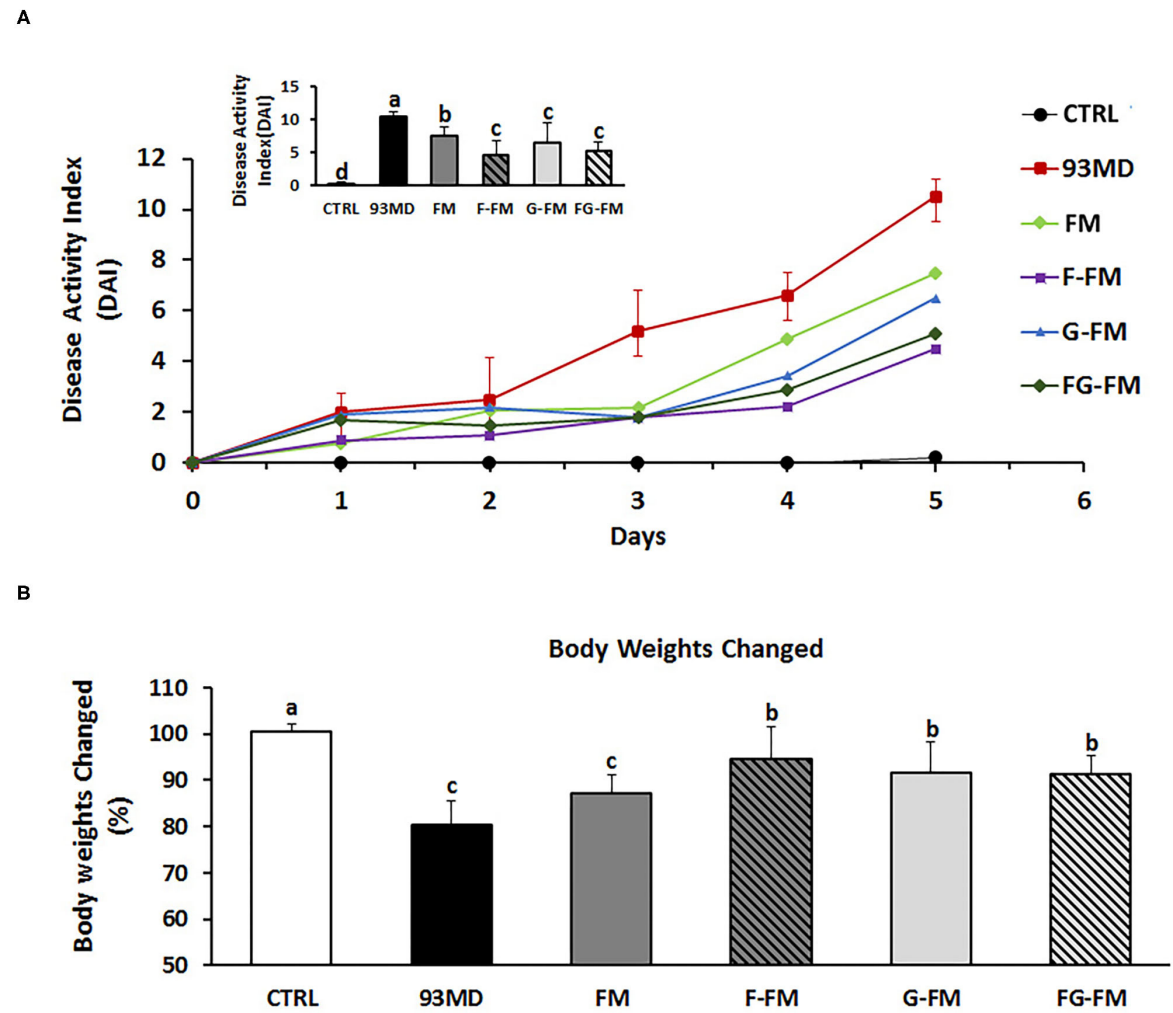
Body physiological: **(A)** DAI (disease activity index) scores monitored every day over the 5 days of DSS exposure; **(B)** relative changes in body weights on day 5 post-induction; comparisons of various anatomical measurements among five groups using one-way analysis of variance (ANOVA), followed by Fisher's LSD *post hoc* tests. Bars with the same letter indicate a non-significant difference (*P* > 0.05). Data are expressed as the mean ±SD. Sample size: *n* = 7 in 93MD group, *n* = 10 in other groups.

After five days of exposure to DSS, all mice exposed to DSS developed certain symptoms of enteritis in comparison to the untreated CTRL group, manifested by significant weight loss, severe diarrhea, and rectal bleeding (Figure 2). As shown in Figure 2A, disease progression in the 93MD group was obviously faster than in the foxtail millet-based diet groups. At the end of DSS induction (Day 5), mice that were fed millet-based diets had a DAI index that was significantly lower than that of the 93MD group, indicating that the four different pre-treated foxtail millet whole grains could relieve the symptoms of enteritis to varying degrees (Figure 2A). From a numerical point of view, the disease index of the two groups fed fermented foxtail millet diets (F-FM and FG-FM) was significantly lower compared to the mice fed unfermented foxtail millet diets (FM and G-FM), although the difference was not significant due to individual variation. In comparison to the 93MD group, the body weight loss of mice from the F-FM, G-FM, and FG-FM groups was alleviated significantly, while the body weight loss of FM showed no significant differences from that of the 93MD group (Figure 2B). These results indicated that both fermentation and germination treatments were effective in alleviating body weight loss during the induction of enteritis in a mouse model.

Moreover, three of the 93MD group mice died at day 5 post-induction due to severe enteritis symptoms. DSS exposure was stopped by the end of day 5 to obtain more data, and the mice were fasted overnight and then dissected the next day. None of the mice in any other groups died during the colitis induction process.

### Pathological Characteristics and Histopathological Scores

**Figure 3:**
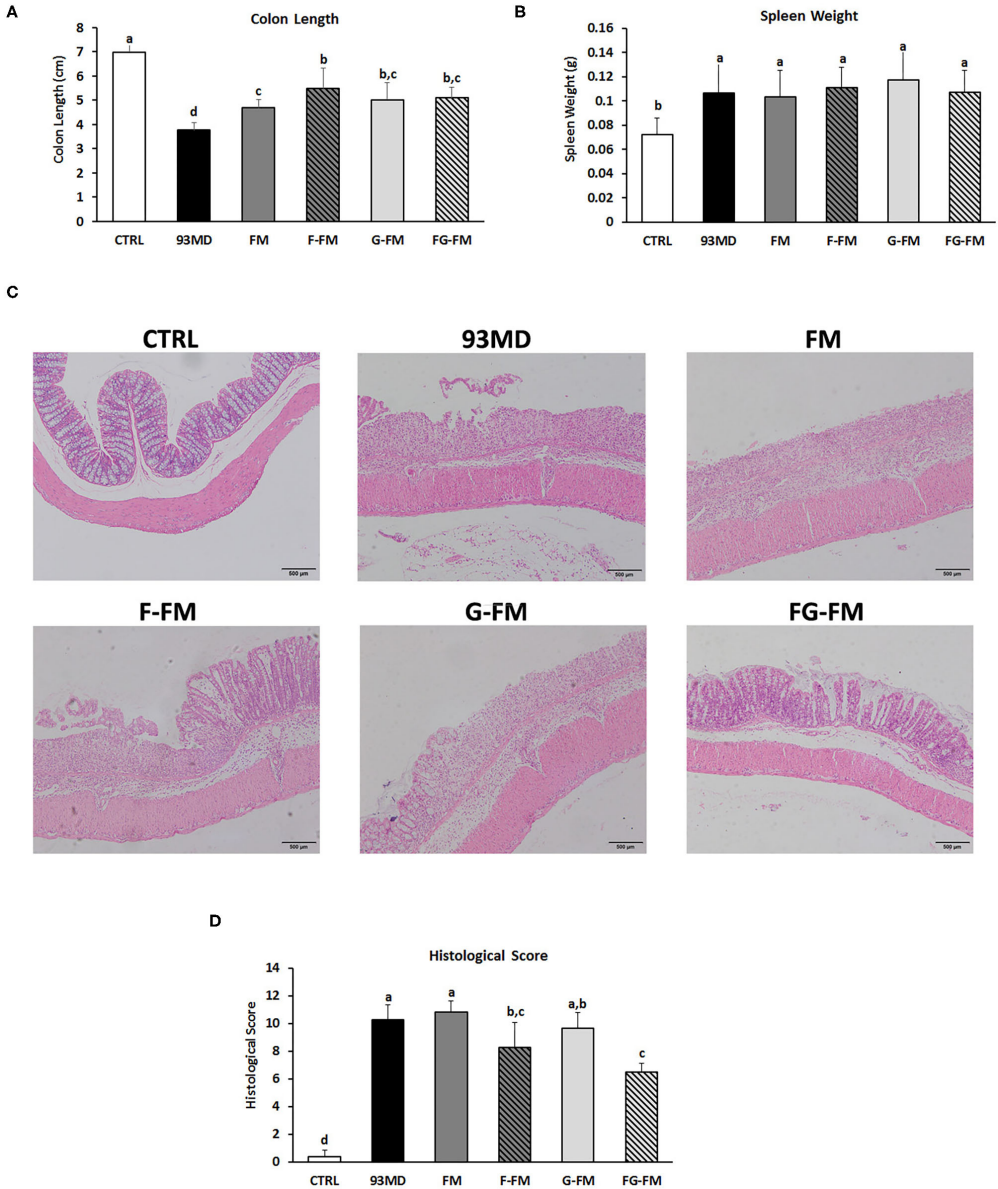
Pathological and histopathological characteristics in different groups: **(A)** lengths of colon from different diet groups; **(B)** spleen weights of the different groups; **(C)** histological analysis and **(D)** histological severity scores of the different treatment mice. Comparisons of various anatomical measurements among six groups using one-way analysis of variance (ANOVA), followed by Fisher's LSD *post hoc* tests. Data are expressed as the mean ± SD. Bars with the same letter indicate a non-significant difference (*P* > 0.05). Sample size: *n* = 7 in 93MD group, *n* = 10 in other groups.

All groups exposed to DSS displayed typical symptoms of enteritis, such as significant shortening of the colon (Figure 3A) and enlarged spleen (Figure 3B), when compared to the control group without DSS treatment. Among the groups exposed to DSS, the shortening of the colon in the 93MD group was the most significant, while in the other groups that were fed foxtail millet-based diets, it was relieved to vary degrees. The shortening of the colon in the FM group was not relieved as well as in the other foxtail millet-based diet groups. There were no significant differences in the spleen weights of the DSS-treated groups fed different diets.

Histopathological examination (Figures 3C,D) of the colon revealed that DSS treatment induced epithelial injury in all eight DSS-treated groups. According to histopathological scores (Figure 3D), mice in the F-FM and FG-FM groups had significantly less intestinal damage than mice in the 93MD group, indicating that the fermentation process was essential to improve the ability of foxtail millet whole grains to protect mice from intestinal injury induced by DSS exposure. When combined with the histological scores of colon tissue damage, DAI, and other indicators, it was concluded that FG-FM was more effective in preventing or alleviating symptoms of colitis.

### Gut Microbial Diversity Indices

**Figure 4:**
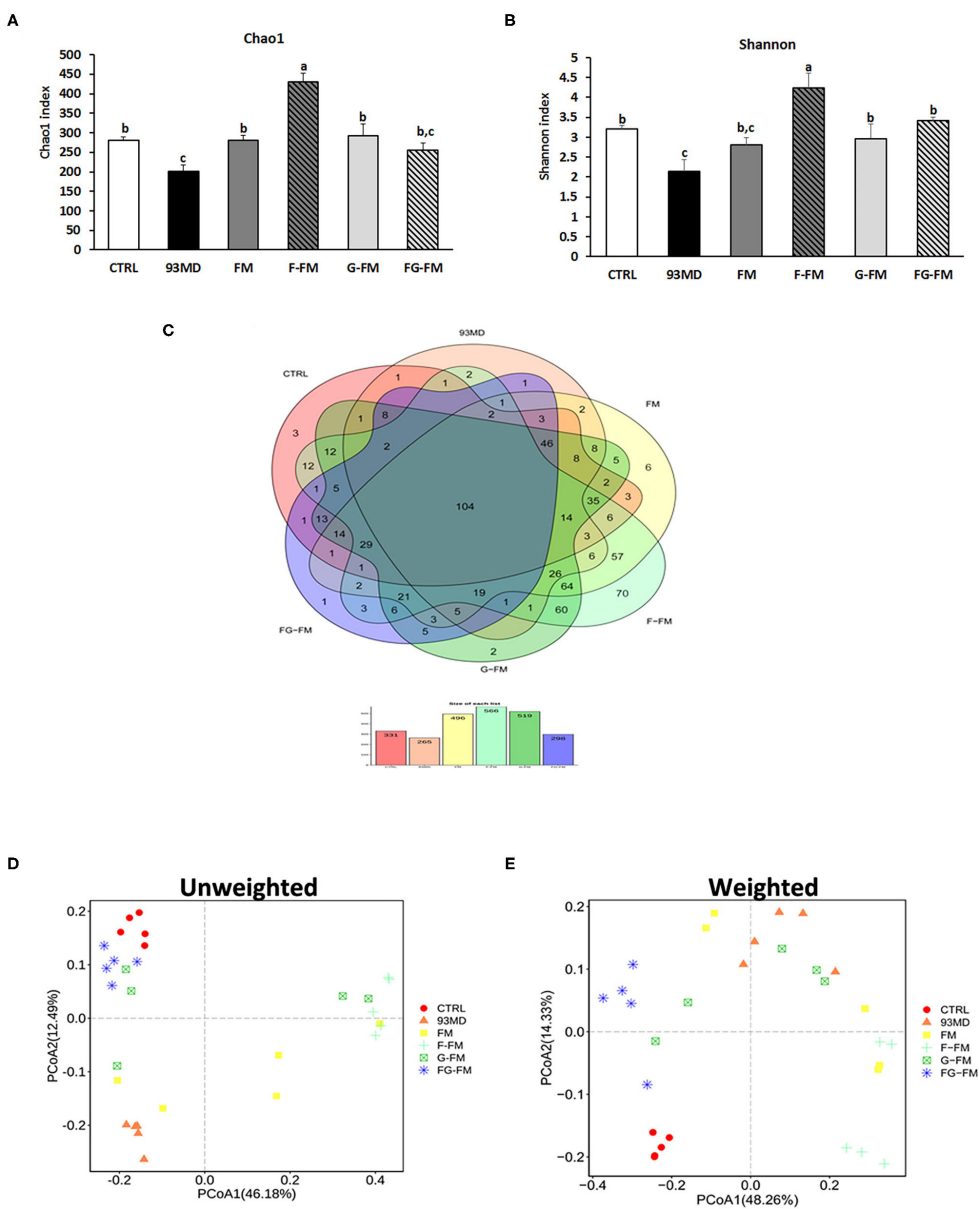
Microbiota diversity indices of the different groups: **(A)** the bacterial richness of microbiota communities estimated by the Chao1 value. **(B)** The bacterial diversity of the microbiota communities estimated by the Shannon index; **(C)** Venn diagrams that illustrated the observed overlap of OTUs; **(D)** PCoA plot of unweighted UniFrac distance values and **(E)** weighted UniFrac distance values. All groups of α diversity indices (Chao1 and Shannon) were analyzed by one-way analysis of variance (ANOVA) followed by Fisher's LSD *post hoc* tests. Bars with the same letter indicate a non-significant difference (*P* > 0.05). Five samples per group were randomly selected for microbiota analysis.

The Chao1 index (Figure 4A) and Shannon diversity index (Figure 4B) were calculated to determine the effects of foxtail millet-based diets on the richness and diversity of bacterial species, respectively. Exposure to DSS induction significantly decreased both the Chao1 index and Shannon diversity index (CTRL vs. 93MD). The FM, F-FM, G-FM, and FG-FM groups had significantly higher Chao1 indices than the 93MD group, indicating that diets made from foxtail millet whole grains significantly restored the bacterial richness of gut microbiota. Shannon indices were similar to Chao 1 indices in terms of details, except that the index of the FM group was not significantly higher than the 93MD group, indicating that foxtail millet whole grains pretreated by germination and fermentation had a stronger ability to restore intestinal microbial diversity than untreated foxtail millet whole grain. The shared and specific OTUs among different groups are represented by Venn diagrams in Figure 4C. A total of 106 OTUs were shared by all six groups, with the F-FM group having the largest number of unique OTUs (seventy). This was consistent with the results of the Chao1 and Shannon indices, which showed that the F-FM group had the highest scores. The improvement of these two indices in the foxtail millet whole grain-based diet group indicated that the foxtail millet whole grain (especially pretreated forms) had a positive effect on the intestinal flora ecosystem. There are reductions in bacterial species richness and diversity in IBD patients (40) and animal models of enteritis (33). Therefore, the promotion of microbial diversity benefits from foxtail millet whole grains may be attributed to the prevention of colitis-related symptoms.

The principal coordinates analysis (PCoA) of weighted and unweighted UniFrac distance matrix of gut microbiota is shown in Figure 4D, (unweighted) Figure 4E (weighted). In both the weighted and unweighted UniFrac plots, the CTRL group showed a significant difference from the 93MD group, as well as clear aggregation with the FG-FM group. In the unweighted UniFrac matrices, neither principal coordinate 1 (PC1, 38.48%) nor PC2 (15.27%) could separate the CTRL group from the FG-FM group; in the weighted UniFrac matrices, the CTRL and FG-FM groups did not separate in PC1 (41.81%) but were barely separated in PC2 (14.15%). This result demonstrated that intake of the FG-FM diet restored the disturbance in gut microbiota composition caused by enteritis to the greatest extent in mouse models. This positive reaction of the structure of gut microbiota communities to FG-FM diets provides important information for further research and development of functional foods for IBD.

### Composition of Gut Microbiota in Different Groups

**Figure 5:**
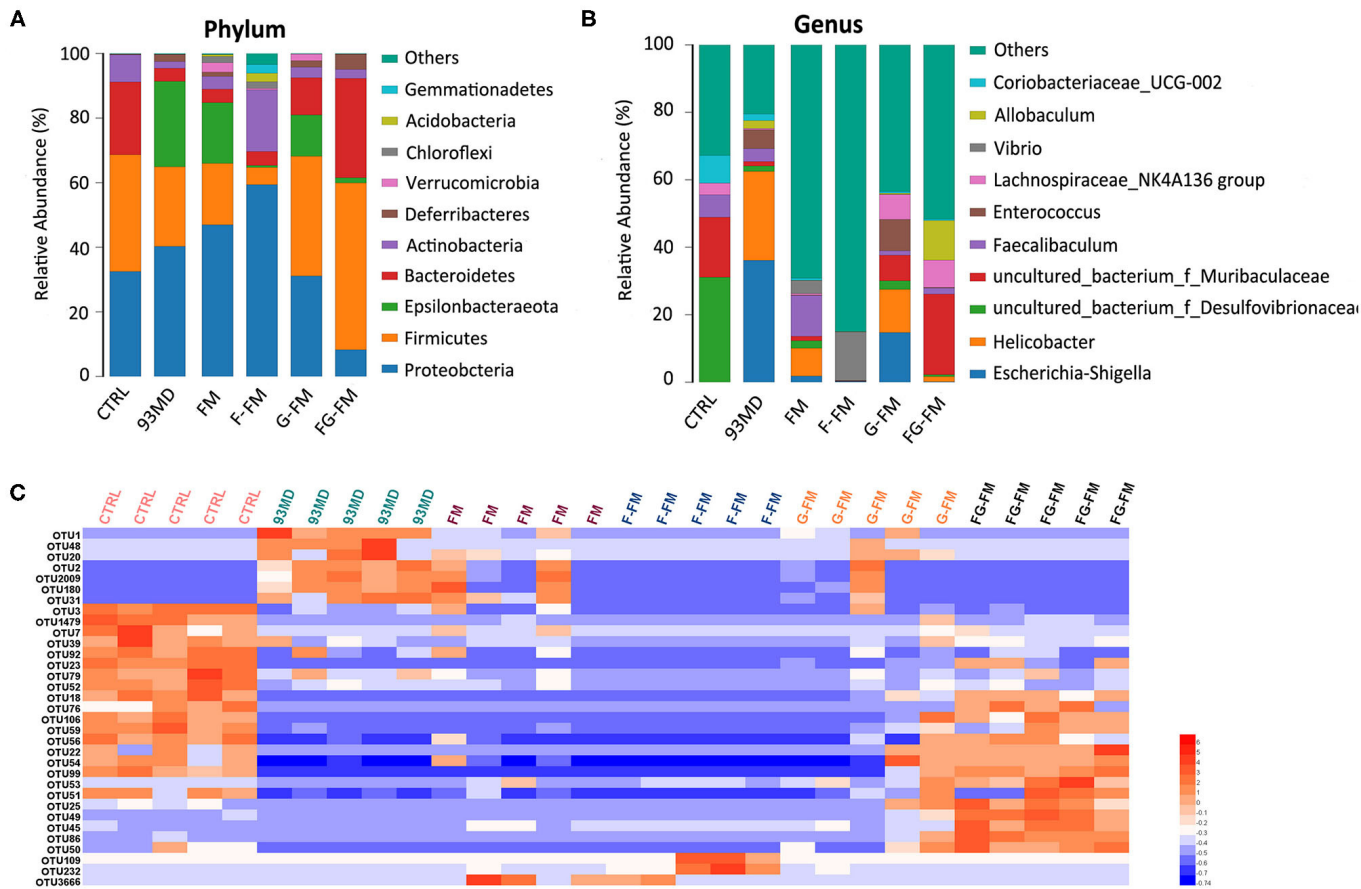
Compositions of gut microbiota in mice at the **(A)** phylum level and **(B)** genus level in all experimental groups and **(C)** heatmap of OTUs (average OTU numbers >100) with significant differences in the gut microbiota of mice (*n* = 5/group).

As illustrated in Figures 5A,B, the composition of gut microbiota varied significantly in mice from different treatment groups. Figure 5A shows the significant variation in the composition of gut microbiota in mice from different groups at the phylum level. In the CTRL group, *Firmicutes* was the most dominant bacterial community (36.1%), followed by *Proteobacteria* (32.5%) and *Bacteroidetes* (22.5%). On the other hand, in the 93MD group, there was a significant reduction in the relative abundance of *Firmicutes* and *Bacteroidetes* (*Firmicutes* = 26.4%; *Bacteroidetes* = 4.1%) and a significant increase in the relative abundance of *Proteobacteria*, which were the most dominant (40.3%). This finding was consistent with the literature, which reported that the unusual expansion of *Proteobacteria* could be considered a “signature” of dysbiosis in gut microbiota (10, 41, 42). It was worth noting that in the four groups that were fed foxtail millet whole grain-based diets, the gut microbiota composition of the mice from the FG-FM group (*Firmicutes*, 51.7%; *Bacteroidetes*, 30.8%; *Proteobacteria*, 8.3%) was close to the ‘normal' state of the CTRL group, which was also consistent with the PCoA results.

The composition analysis of the gut microbiota at the genus level (Figure 5B) revealed dramatic blooms of the genera *Escherichia-Shigella* and *Helicobacter* in the 93MD group (*Escherichia-Shigella*, 36.1%; *Helicobacter*, 26.4%), which were almost undetectable in the CTRL group. *Escherichia-Shigella* even occupied a predominant position in the gut microbiota of the 93MD group. However, the overexpansion of *Escherichia-Shigella* and *Helicobacter* was almost completely suppressed in the gut microbiota of mice from the F-FM and FG-FM groups.

The significant OTUs (operational taxonomic units) associated with DSS induction and diet intervention were also analyzed (Figure 5C). The corresponding detailed data, statistical results, and classification information are presented in Supplementary Material. As shown in Figure 5C, the numbers of OTU2, OTU1, OTU48, OUT20, OTU2, OTU2009, OTU180, and OTU31 increased in the 93MD group (compared to the CTRL group), whereas the increasing trend was inhibited to varying degrees in the foxtail millet whole-grain diet groups. These OTUs were classified as uncultured bacteria from the genera OTU2 *Helicobacter* (OTU2), *Escherichia-Shigella* (OTU1), *Allobaculum* (OTU48), *Coriobacteriaceae_UCG-002* (OTU20), *Mucispirillum* (OTU2009 and OTU180), and *Dubosiella* (OTU31). Unlike *Helicobacter* and *Escherichia-Shigella*, the genera *Allobaculum, Coriobacteriaceae_UCG-002, Mucispirillum*, and *Dubosiella* could not be classified as either pro-inflammatory or anti-inflammatory bacteria. Taking the genus *Mucispirillum* as an example, it was identified to be associated with inflammatory bowel disease (43) or other inflammatory diseases (44), whereas it was also reported to be related to a healthier state of gut microbiota (45). These seemingly contradictory conclusions also reflect the complexity of gut microbiota structure and function.

According to Figure 5C, there were 16 OTUs that obviously decreased in the 93MD group compared to the CTRL group, and six of these OTUs (including OTUs 56, 22, 54, 76, 99 and 106) showed the same characteristics; that is, the numbers of these six OTUs showed an obvious rebond in the FG-FM group. These six OTUs were classified into uncultured bacterial strains from the genus *Lachnoclostridium* (OTU106), genus *Desufovibrio* (OTU22), and *Lachnospiraceae NK4A136* group (OTU76) and three uncultured bacterial strains from the family *Muribaculaceae* (OUT54, OTU56, and OTU99). Moreover, there were 5 OTUs significantly higher in the FG-FM group than in the other five groups, which were classified as species *Mucispirillum schaedleri* ASF457 (OTU25), uncultured bacteria from the genus *Lactobacillus* (OTU45), genus *Alistipes* (OTU50), and family *Muribaculaceae* (OTU49 and OTU86). Five OTUs classified to the family *Muribaculaceae* were significantly higher in the FG-FM group, which was consistent with the predominant position of *Muribaculaceae* in the gut microbiota of the FG-FM group (demonstrated in Figure 5B).

### Plasma Levels of Inflammatory Cytokines and Colonic Barrier Function

**Figure 6:**
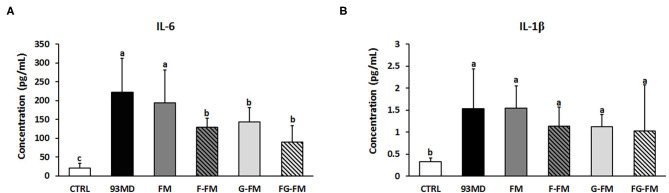
Levels of inflammatory cytokines in the plasma of mice, **(A)** IL-6; **(B)** IL-1β. All data were analyzed by one-way analysis of variance (ANOVA) followed by Fisher's LSD *post-hoc* tests. Bars with the same letter indicate a non-significant difference (*P* > 0.05). Sample size: *n* = 7 in 93 MD group, *n* = 10 in other groups.

The levels of the inflammatory cytokines IL-6 and IL-1β in the plasma are shown in Figure 6. DSS exposure increased the circulating levels of IL-6 and IL-1β compared to the untreated CTRL group. Certain foxtail millet whole grain-based diets reduced the levels of IL-6, with the FG-FM group showing the most potent anti-inflammatory effects.

As demonstrated in Table 4, the expression levels of two key tight junction proteins, *ZO-1* and *occludin*, were dramatically decreased in the DSS-induced colitis mice compared to the CTRL group. The loss of *ZO-1* and *occludin* genes has been reported in DSS-induced colitis mouse models (46, 47). The expression of *ZO-1* and *occludin* was also below the detection level in the 93MD group. However, it was partially restored in the foxtail millet-based diet-fed mice, especially *ZO-1* in the FG-FM group. Unlike *ZO-1* and *occludin*, the expression of *claudin 1* and *claudin 2* was significantly upregulated in the majority of DSS-induced colitis mice when compared to the CTRL group without DSS treatment. This observation was consistent with previous studies that reported the upregulation of *claudin 1* and *claudin 2* increased intestinal permeability and aggravated inflammation (48, 49). The intake of the FG-FM diet significantly inhibited the overexpression of *claudin2*, which also indicated the maintenance of gut barrier function in this group.

**Table 4:** Expression levels of genes related to gut barrier function in the mouse colon.

**Groups**	***Claudin1***	***Claudin2***	***ZO-1***	***Occludin***
**CTRL**	1.00 ± 0.43 **(b)**	1.00 ± 0.44 **(b, c)**	1.00 ± 0.27 **(a)**	1.00 ± 0.079 **(a)**
**93MD**	10.97 ± 8.16 **(a)**	1.53 ± 0.23 **(a)**	ND^*^	ND^*^
**FM**	8.49 ± 3.31 **(a)**	1.29 ± 0.61 **(a, b)**	0.020 ± 0.025 **(b)**	0.0052 ± 0.0043 **(b)**
**F-FM**	7.69 ± 2.92 **(a)**	1.19 ± 0.39 **(a, b)**	0.29 ± 0.37 **(b)**	0.016 ± 0.022 **(b)**
**G-FM**	8.03 ± 4.62 **(a)**	1.21 ± 0.27 **(a, b)**	0.25 ± 0.44 **(b)**	0.0017 ± 0.0015 **(b)**
**FG-FM**	6.49 ± 2.71 **(a)**	0.69 ± 0.12 **(c)**	0.52 ± 0.88 **(a)**	0.018 ± 0.011 **(b)**

*The data are expressed as the relative fold change compared to the CTRL group*.

*Data were expressed as Mean ± SD*.

*The same letter (Bold) indicates a non-significant difference (P > 0.05)*.

* *Indicated below the detection limit (non-detectable)*.

## Discussion

Despite continuous efforts, the existing drugs and treatments are still not completely effective for the treatment and prevention of IBD, and pharmacological side effects of drugs are usually inevitable (50). Therefore, there is an increasing demand for the development of effective prevention strategies for IBD. Dietary strategies are very attractive in the prevention of chronic inflammation-related diseases, especially IBD, due to their low cost and safety (51). Foxtail millet is a traditional staple food in the northern areas of China and is mainly consumed as porridge. In this study, it was demonstrated that foxtail millet whole grain supplementary diets were suitable for dietary intervention in IBD. The germination or fermentation process optimized the disease suppression ability of whole-grain foxtail millet. Furthermore, whole grain foxtail millet that had undergone dual processing of germination and fermentation showed excellent anti-inflammatory properties and prebiotic characteristics, which makes it not only effective for IBD patients but also has the potential to be used as an approach to treat or prevent other intestinal diseases.

When the foxtail millet was consumed as whole grain without debranning and polishing, the fermentation process significantly enhanced its prebiotic characteristics and function to alleviate the symptoms of colitis. When consumed as a whole grain, it introduced higher dietary fiber to the diet. Although dietary fibers have been reported to possess clinical benefits for IBD patients (52), the untreated whole grain foxtail millet had no effect on relieving the symptoms of colitis in this study. However, whole grain foxtail millet that had been germinated or fermented, especially those that had undergone both the germination and fermentation processes (FG-FM group), could relieve the symptoms of enteritis to varying degrees.

The analysis of compounds in different pre-treated foxtail millet could also partially explain why the fermentation or germination process could significantly improve colitis symptoms relief and its prebiotic abilities. Despite the availability of various compounds and that most of them have not been extensively studied, the relationships between several compounds and IBD have been established. For example, nicotinamide, which was up-regulated via both fermentation and germination process, have been proved to contribute to the amelioration of experimental colitis (53). A similar case is the compound N-methyltyramine (54), although there is no direct evidence to prove its beneficial effect on IBD, a recent study has begun to focus on its role in intestinal health. Due to the complexity of the changes in the chemical composition of foxtail millet whole grain caused by fermentation or germination, the more attention should focus on this area.

The FG-FM flour displayed excellent prebiotic characteristics, including restoration of the overall composition of the intestinal flora to near-normal levels, inhibition of the proliferation of the conditional pathogenic bacteria, and promotion of beneficial bacteria growth. Although the underlying mechanism is far to be fully understood, there is no doubt that the gut microbiota plays a vital role in the pathogenesis and development of IBD (6, 10). This can also explain why FG-FM was effective in relieving the symptoms of colitis to a certain extent. For instance, *Escherichia-Shigella*, which was prevalent in the IBD patients (55), and also had higher relative abundance in the DSS-induced colitis model (56), was almost completely suppressed in the FG-WFM diet-fed mice. The predominant position of *Muribaculaceae* in the gut microbiota of the FG-FM group was also observed. In most studies, *Muribaculaceae* (also known as S24-7 clade or *Candidatus Homeothermaceae*) was significantly decreased in disease models (such as obesity, diabetes, and intestinal colitis) and rebonded fowllowing effective treatment or dietary intervention (57–59). Therefore, an increase in *Muribaculaceae* should be considered as a marker of the recovery of gut microbiota. In addition, the growth stimulation of probiotics, such as *Lactobacillus* (56), may be associated with the alleviation of colitis symptoms in the FG-FM group. This evidence indicated that the prebiotic characteristics of FG-FM might contribute to the alleviation of the symptoms of colitis in mouse models.

The foxtail millet whole grain-based diet (FG-FM) also displayed efficient anti-inflammatory activity in DSS-induced colitis mouse models. The rebalancing ability of FG-FM on gut microbiota contributed to its excellent anti-inflammatory activity based on the reported role of gut microbiota in inflammation (6). Functional loss of the gut barrier and a decrease in gut permeability are also characteristics of IBD. The upregulation of *ZO-1* and *occludin* expressions, as well as the downregulation of *claudin 2* expression, indicated that FG-FM consumption significantly increased the function of the gut barrier. Furthermore, a study reported that IL-6 monoclonal antibody treatment using DSS-induced colitis mouse models effectively suppressed the expression of *claudin 2* and attenuated gut permeability (60). This finding suggested that in FG-FM diet-fed mice, a decrease in inflammatory cytokine levels is correlated with an improved gut barrier function.

In summary, this study demonstrated that the foxtail millet supplementary diets are suitable for dietary intervention in IBD patients based on a mouse model. The germination or fermentation process optimizes the disease suppression ability of whole-grain foxtail millet. Moreover, whole grain foxtail millet that has undergone dual processing of germination and fermentation had excellent pro-inflammatory activity and prebiotic characteristics, making it effective not only for IBD patients but also for the treatment or prevention of other intestinal diseases.

## Data Availability Statement

The datasets presented in this study can be found in online repositories. The names of the repository/repositories and accession number(s) can be found below: https://www.ncbi.nlm.nih.gov/, PRJNA719303.

## Ethics Statement

The animal study was reviewed and approved by Ethics Committee of the Institute of Agro-food Science and Technology at Shandong Academy of Agricultural Sciences (Jinan, Shandong, China).

## Author Contributions

WL: conceptualization, supervision, formal analysis, writing-original draft, and writing-review and editing. LL: conceptualization and writing-review and editing. YZ: investigation and writing-original draft preparation. DZ: supervision and investigation. XW: investigation. YY: resources. All authors contributed to the article and approved the submitted version.

## Conflict of Interest

The authors declare that the research was conducted in the absence of any commercial or financial relationships that could be construed as a potential conflict of interest.

## Publisher's Note

All claims expressed in this article are solely those of the authors and do not necessarily represent those of their affiliated organizations, or those of the publisher, the editors and the reviewers. Any product that may be evaluated in this article, or claim that may be made by its manufacturer, is not guaranteed or endorsed by the publisher.
