# Supplementary material for: Fermented and Germinated Processing Improved the Protective Effects of Foxtail Millet Whole Grain Against Dextran Sulfate Sodium-Induced Acute Ulcerative Colitis and Gut Microbiota Dysbiosis in C57BL/6 Mice
Source: Front Nutr. 2021 Jul 29;8:694936. doi: 10.3389/fnut.2021.694936 (PMC8358663; doi:10.3389/fnut.2021.694936)
Supplement: Supplementary file 1 [file Data_Sheet_1.docx]

Supplementary Material

Fermented and germinated processing improved the protective effects of foxtail millet whole grain against dextran sulfate sodium-induced colitis and gut microbiota dysbiosis in C57BL/6 mice

**Supplementary Table 1: The macronutrients compositions of cereal flours**

| **Nutrients** | **FM** | **F-FM** | **G-FM** | **FG-FM** |
| --- | --- | --- | --- | --- |
| Starch (%) | 58.70 | 61.11 | 59.92 | 58.54 |
| Protein (%) | 9.80 | 10.74 | 11.14 | 10.87 |
| Fat (%) | 3.69 | 3.65 | 3.64 | 2.85 |
| Dietary Fiber (%) | 17.24 | 17.10 | 17.06 | 19.41 |
| Moisture (%) | 7.84 | 4.54 | 5.56 | 5.47 |
| Ash (%) | 2.74 | 2.88 | 2.70 | 2.87 |

**Supplementary Table 2: Composition of experimental diets used in this study**

| **Composition** | **Diet (g/1000 g)** | | | | |
| --- | --- | --- | --- | --- | --- |
|  | **AIN-93M** | **FM** | **F-FM** | **G-FM** | **FG-FM** |
| Casein | 140 | 91.02 | 96.32 | 84.30 | 85.65 |
| L-Cystine | 1.8 | 1.8 | 1.8 | 1.8 | 1.8 |
| Corn starch | 495.69 | 94.67 | 89.37 | 101.39 | 100.04 |
| Refined wheat flour | 0 | 0 | 0 | 0 | 0 |
| Differential pre-treated foxtail millet flour | 0 | 500 (FM) | 500 (F-FM) | 500 (G-FM) | 500 (FG-FM) |
| Maltodextrin | 125 | 125 | 125 | 125 | 125 |
| Sucrose | 100 | 100 | 100 | 100 | 100 |
| Cellulose | 50 | 0 | 0 | 0 | 0 |
| Soybean Oil | 40 | 40 | 40 | 40 | 40 |
| Mineral mixture(AIN-93M-MX)* | 35 | 35 | 35 | 35 | 35 |
| Vitamin mixture(AIN-93-VX)* | 10 | 10 | 10 | 10 | 10 |
| Bitartrate Choline | 2.5 | 2.5 | 2.5 | 2.5 | 2.5 |
| Tert-butylhydroquinone | 0.008 | 0.008 | 0.008 | 0.008 | 0.008 |
| Total | 1000 | 1000 | 1000 | 1000 | 1000 |

The compositions of mineral and vitamin mixture were followed the description of Reeves et al., 1993.

**Supplementary Table 3: Primers for detection of relative abundance of genes related to gut barrier function in mouse colon**

| **Gene Name** | **Forward Primer** | **Reverse Primer** |
| --- | --- | --- |
| *Claudin 1* | GGGGACAACATCGTGACCG | AGGAGTCGAAGACTTTGCACT |
| *Claudin 2* | CAACTGGTGGGCTACATCCTA | CCCTTGGAAAAGCCAACCG |
| *ZO-1* | GCCGCTAAGAGCACAGCAA | TCCCCACTCTGAAAATGAGGA |
| *occludin* | TGAAAGTCCACCTCCTTACAGA | CCGGATAAAAAGAGTACGCTGG |
| *GAPDH* | TTGATGGCAACAATCTCCAC | CGTCCCGTAGACAAAATGGT |
